# A Case of Debilitating Abdominal Pain: Gastric Twist Post Sleeve Gastrectomy

**DOI:** 10.7759/cureus.90282

**Published:** 2025-08-17

**Authors:** Radwan Elmasri, Bassel Hamam, Sarah Jalloul, Ibtihaj Saad, Johny Salem, Walid Nassredine

**Affiliations:** 1 Internal Medicine, Balamand University, Beirut, LBN; 2 Radiology, Balamand University, Beirut, LBN; 3 Gastroenterology and Hepatology, Balamand University, Beirut, LBN

**Keywords:** gastric twist, gastropexy, roux-en-y gastric bypass alternative, sleeve gastrectomy, stenosis

## Abstract

Gastric twist (GT) is a rare and often underdiagnosed condition that requires a high index of suspicion due to its potential to cause significant morbidity if not promptly recognized and managed. Diagnosis typically begins with an upper gastrointestinal (GI) contrast study, which is particularly useful for visualizing the orientation and rotation of the stomach. Endoscopy is usually performed afterward to rule out intraluminal pathology and, in some cases, to confirm the degree of torsion. In select situations, computed tomography (CT) may help identify associated anatomical abnormalities or complications. Management depends on symptom severity and anatomical findings. Medical therapy, including acid suppression and dietary modification, is generally reserved for patients with mild or intermittent symptoms. Endoscopic management, such as metallic stenting, may be attempted in cases of distal sleeve stenosis. However, surgical intervention is often required for patients with persistent, severe, or recurrent symptoms. We report a case of GT diagnosed 10 years after laparoscopic sleeve gastrectomy (LSG), successfully treated with ligamentum teres cardiopexy, resulting in complete symptom resolution.

## Introduction

Laparoscopic sleeve gastrectomy (LSG) promotes weight loss primarily through restriction by reducing gastric volume, and secondarily through hormonal mechanisms, including suppression of ghrelin and increased secretion of GLP-1 secondary to a rise in L cells [1]. LSG has shown excellent weight loss outcomes and significant improvement in obesity-related comorbidities, often matching or surpassing other established bariatric procedures [2].

Evidence from 36 studies involving 2,570 patients indicates that LSG is a safe and effective option, whether performed as a primary or staged procedure, with sustained weight loss and comorbidity reduction comparable to other bariatric surgeries [2].

Functional stenosis, also referred to as gastric twist (GT), represents a rare late complication of LSG, with only a few cases reported years after surgery.

## Case presentation

A 55-year-old woman with a history of dyslipidemia, epilepsy (diagnosed 27 years prior), cluster headaches, iron deficiency anemia, and obesity (status post sleeve gastrectomy 10 years earlier) presented with worsening abdominal symptoms. She had also undergone a cholecystectomy six months earlier. The recent cholecystectomy, in the context of prior sleeve gastrectomy, raised concern for adhesions or altered anatomy contributing to her symptoms, including the possibility of GT.

Her symptoms began in February 2021, three years before her current presentation, with epigastric pain that gradually became diffuse, postprandial, and non-radiating, leading to poor oral intake. The pain was consistently relieved by non-bloody, non-bilious vomiting. She reported persistent gastroesophageal reflux disease (GERD) symptoms despite medical therapy, chronic constipation requiring laxatives, and an unintentional 20-kg weight loss over the past year. The severity and frequency of her symptoms increased, resulting in multiple emergency department visits, hospital admissions, and opioid use for pain control.

Initial laboratory findings were notable only for anemia. Abdominopelvic computed tomography (CT) with intravenous (IV) contrast was unremarkable. Given her worsening symptoms and negative CT results, advanced MRI protocols (magnetic resonance angiography (MRA), magnetic resonance venography (MRV), magnetic resonance cholangiopancreatography (MRCP)) were performed to rule out mesenteric ischemia, vascular abnormalities, and biliary or pancreatic duct pathology, but no significant abnormalities were detected.

**Figure 1:**
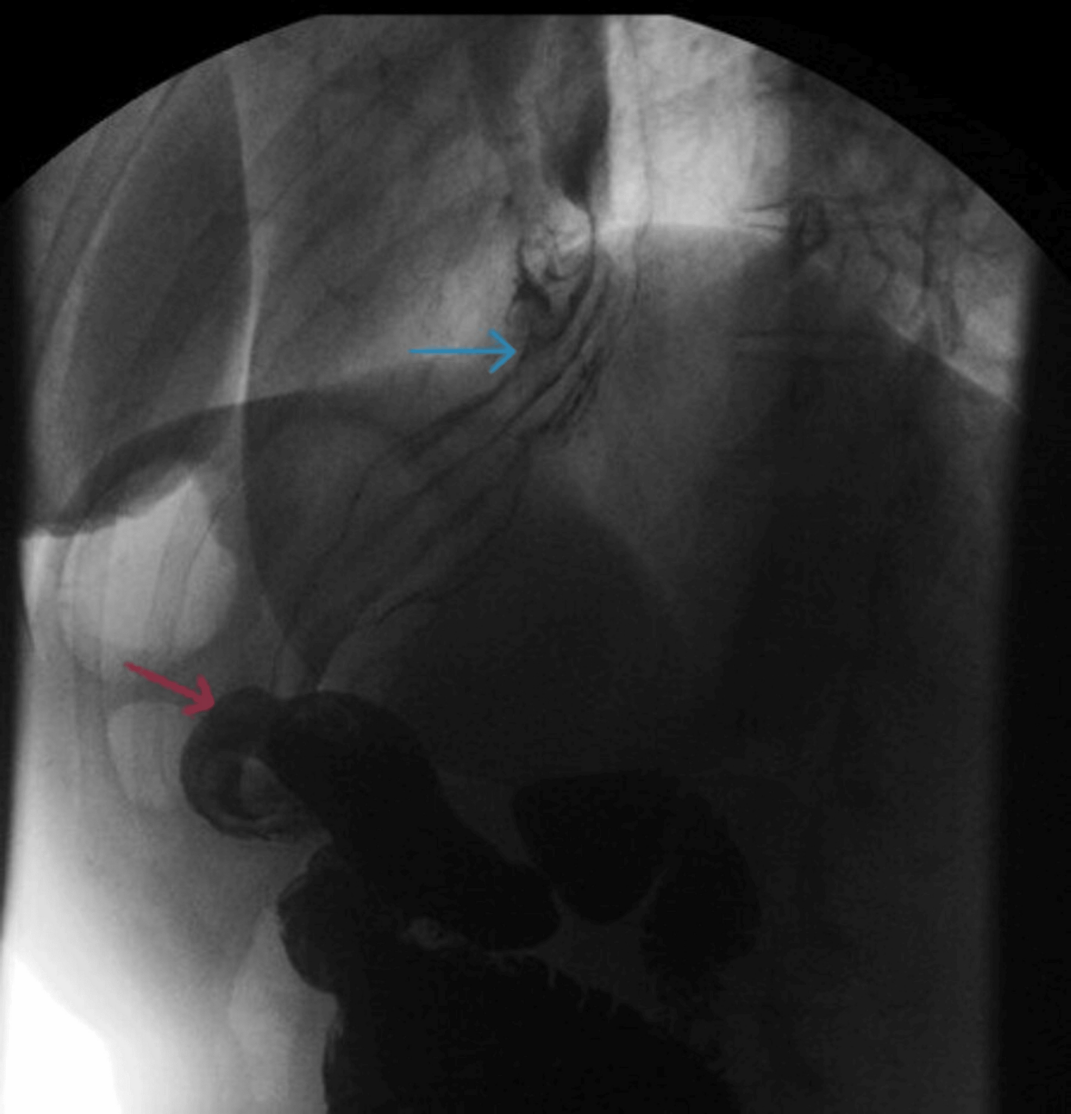
Upper gastrointestinal series showing gastric twist Barium swallow demonstrates gastric twisting and angulation (red arrow) with a moderate sliding hiatal hernia (blue arrow).

An esophagogastroduodenoscopy (EGD) was subsequently performed to evaluate for gastric outlet obstruction. This revealed a small hiatal hernia and Grade B esophagitis, but no strictures or obstructive lesions. However, advancing the scope into the antrum proved difficult due to stiff angulation. This technical challenge suggested functional angulation, which was later confirmed as GT on an upper GI (UGI) contrast study. Biopsies were negative for *H. pylori*, intestinal metaplasia, or dysplasia. The UGI series demonstrated a moderate sliding hiatal hernia with distal GT and angulation, without evidence of leakage (Figure 1).

Due to persistent symptoms and progressive weight loss, the multidisciplinary team opted for ligamentum teres cardiopexy rather than Roux-en-Y gastric bypass (RYGB), given its lower risk of further weight loss and malabsorption. Intraoperative findings included a hiatal hernia, adhesions along the greater curvature and staple line, and GT. Surgical management involved hiatal hernia repair, round ligament gastropexy, crural closure, and adhesiolysis.

Postoperatively, the patient was monitored for 48 hours, tolerated oral intake, and was discharged on postoperative day 3, symptom-free and off analgesics. At six-month follow-up, she reported complete symptom resolution, though long-term surveillance is ongoing.

## Discussion

GT is a rare but clinically significant late complication of LSG, reported in approximately 0.7%-4% of cases [3], and up to 10% following revisional bariatric surgery [4]. Symptoms are often nonspecific and frequently misdiagnosed, as in our patient who was initially labeled with severe irritable bowel syndrome (IBS). Abdominal pain, nausea, vomiting, and GERD symptoms are common to both IBS and GT, making diagnosis particularly challenging in the absence of overt obstruction or abnormal findings on routine endoscopy. While some patients with GT remain asymptomatic, others may present with varying degrees of nausea, vomiting, reflux, or abdominal pain depending on the severity of the twist [5], all of which were evident in our case.

Gastric stenosis may be classified as either organic or functional, with GT more closely aligned to the functional type. The exact mechanism of twist formation is not fully understood, but three main theories are described in the literature: (1) fixation of part of the stomach by surrounding ligaments, (2) asymmetric traction on the anterior and posterior walls during stapling of the sleeve, and (3) sleeve scarring with adhesion formation leading to kinking of the gastric tube [6]. In our case, intraoperative findings demonstrated dense adhesions and kinking, consistent with the third mechanism. Progressive scarring and adhesion formation likely contributed to the delayed onset and nonspecific nature of the patient’s symptoms.

Diagnosis of GT typically requires a UGI series to confirm the presence of functional stenosis. Contrast-enhanced CT with oral and IV phases, along with three-dimensional reconstruction, may serve as an adjunct in cases where the UGI series is non-diagnostic or equivocal. EGD is also a valuable diagnostic tool and can be therapeutic in selected cases. In a series of 90 patients reported by Siqueira et al., 91.1% had degree I GT, 6.7% degree II, and 2.2% degree III [7]. Most patients were asymptomatic, but among those with symptoms, vomiting was the most common clinical manifestation.

The absence of significant anatomical obstruction, combined with the patient’s chronic, non-specific symptoms, suggests she most likely had a degree II GT. This is typically characterized by moderate rotation of the staple line that requires additional endoscopic maneuvers to traverse. Although degree II twists can sometimes be managed conservatively, her persistent symptoms and lack of response to medical therapy made surgical intervention necessary. 

Not only did the article shed light on the endoscopic classification, but it also highlighted its role in guiding management decisions. Patients with degree I twists were treated successfully with proton pump inhibitors, while degree II and III twists required more invasive approaches, such as balloon dilation with fully covered self-expandable metal stents (particularly in cases with leaks) or conversion to RYGB [8].

This case is unusual, as GT typically presents within the first postoperative year, whereas our patient developed symptoms 10 years after LSG. Given the absence of obstruction on EGD and her persistent, debilitating symptoms, surgical management was favored over endoscopic approaches.

Ligamentum teres cardiopexy was chosen instead of RYGB because the patient had already lost approximately 20 kg in the past year. The procedure stabilizes the stomach by securing the ligamentum teres, reducing the risk of recurrent twisting or volvulus, while preserving gastric anatomy and function. In contrast, RYGB carries higher morbidity and risk of malabsorption, which would have been detrimental in a patient already experiencing significant weight loss.

## Conclusions

This case of GT following LSG illustrates the diagnostic challenges and underscores the need for a high index of suspicion and a multidisciplinary approach to achieve timely and accurate diagnosis. While most studies recommend definitive surgical intervention, such as RYGB or gastropexy, when conservative management fails, the choice of ligamentum teres cardiopexy in this case was guided by the patient’s nutritional status, with a 20-kg weight loss and a BMI of 19 kg/m², while also aiming to minimize the morbidity associated with Roux-en-Y. Ultimately, this case highlights that management must be individualized, and further studies are warranted to refine treatment strategies and improve understanding of the underlying pathophysiology.
